# MTT assay based inhibition analysis of A2780 cells proliferation using *Mollugo nudicaulis* Lam. extract with CXCR4 and HER2 expression

**DOI:** 10.6026/97320630017705

**Published:** 2021-07-31

**Authors:** Chella Perumal Palanisamy, Shazia Fathima JH, Ramya Sekar, Ponnulakshmi Rajagopal, Selvaraj Jeyaraman

**Affiliations:** 1 State Key Laboratory of Biobased Material and Green Papermaking, School of Food Science and Engineering, Qilu University of Technology, Shandong Academy of Science, Jinan 250353, China; 2 Department of Oral and Maxillofacial Pathology, Ragas Dental College and Hospitals, Chennai, India; 3 Department of Oral Pathology, Meenakshi Ammal Dental College, Chennai-600 095, India; 4 Central Research Laboratory, Meenakshi Academy of Higher Education and Research (Deemed to be University), Chennai-600 078, India; 5 Department of Biochemistry, Saveetha Dental College and Hospitals, Saveetha Institute of Medical and Technical Sciences, Saveetha University, Chennai - 600 077, India

**Keywords:** *M. nudicaulis*, n-hexane extract, CXCR4 and HER2 protein expression, Metastatic signalling, Anti-ovarian cancer activity

## Abstract

It is of interest to document the inhibition of A2780 cell proliferation using *Mollugo nudicaulis* Lam.(*M.nudicaulis*) extract by MTT assay and by monitoring the CXCR4 and HER2 expression through RT-PCR analysis. Results shown that the n-hexane extract of *M.nudicaulis* have anticancer activity IC_50_ values of 32.46±0.92 µg/mL on A2780 cell lines. It is further found that the CXCR4 and HER2 mRNA and protein expression were significantly reduced in *M.nudicaulis* treated A2780 cell lines. Thus, the n-hexane extract of *M.nudicaulis* is a natural source of bioactive compounds as potential anticancer agents.

## Background:

In the current scenario, research and development (R&D) in pharmaceutical industries emphasize finding the superordinate actions intended to deal with the intervention of cancer and therapeutic agent counteracting force [1]. Cancer, a dreadful lethal disease in high-income countries, and is at the No. 1 position surpassing heart disease, which emphasizes the crucial requisite to change treatment and prevent this disease's approach [2]. Available FDA approved cancer drugs (such as alkylating agents, platinum compounds, and antimetabolites) may also cause cytotoxicity [3]. DNA mutations were considered as drivers and may not have a role in tumor initiation, however by discerning the role of these mutations would pave way in designing an effective drug to confront the disease [4 - check with author]. The World Health Organization's (WHO) exclusive antibiotic resistance is a greater unsatisfactory situation since 1994 due to the lack of new antibiotics classes. Hence, beneficial and or less harmful treatments are needed, which is only possible when combined with the natural products obtained from the medicinal plants [5].

The frequent use of the synthetic drug leads to cause a variety of side effects and occasionally drug resistance [6 - check with author]. Unlike a synthetic drug, natural products possess significant activities in the controlling and preventing various diseases and disorders without causing unwanted side effects [7]. Hence, searching for new natural products prepared from medicinal plants is subject to this issue [8 - check with author]. *Mollugo nudicaulis* Lam. (*M.nudicaulis*) is one of the Indian medicinal plants which belongs to Molluginaceae family [9]. Traditionally, it is used in Indian phytotherapy to treat wounds, cough, cold, fever, inflammation, cancer, urinary and kidney infections [10]. Ethanolic extract of M .nudicaulis leaves possesses various phytochemicals like alkaloids, flavonoids, terpenoids and other phenolic compounds, and it is also exhibited anti-diabetic, anti-inflammatory activities in an animal model [11]. Data on the anti cancer activity of the n-hexane extract of *M.nudicaulis* is not known. Therefore, it is of interest to document the inhibition of A2780 cell proliferation using *M.nudicaulis* Lam extract by MTT assay and by monitoring the CXCR4 and HER2 expression through RT-PCR data.

## Materials & Methods:

### Plant collection and authentication

The whole fresh plant of *M. nudicaulis* was gathered from Keeranur, Pudukkottai District, Tamil Nadu, India, and it was established as genuine by Dr. G.V.S. Murthy, Botanical Survey of India, Tamilnadu Agricultural University Campus, Coimbatore, Tamil Nadu (Reference: BSI/SRC/5/23/10-11/Tech 420), then the plant materials were dried under the shade condition, pulverized and stocked in air tight jar at 4°C for future analysis [12].

### Extract preparation

The plant material of *M. nudicaulis* was obtained by the method of exhaustive extraction [13 - check with author]. Briefly, 300g of the plant material was soaked in 1500 mL of n-hexane contained flask and placed on a rotating shaker for 72 hrs at 25°C (average room temperature). Finally, the collected infusion was concentrated through a rotary evaporator (RE-2A evaporator) set at 40°C. Further, it was stored at 4°C for future studies.

### Antiovarian cancer activity

Cell growth suppression was found by MTT assay [14]. Briefly, 5000 cells were seeded in each well in ninety six-well plates and cultured for twenty-four hours; as a next step the assay treated with different concentration (3.12, 6.25, 12.5, 25, 50, 100, 200 µg/mL) of plant extract while cyclophosphamide was employed as an optimistic check. The cells were then incubated for twenty-four hrs at thirty seven degree celsius in 5% CO_2_. Towards the end of incubation, the medium was secluded and 10 µL of MTT was added proceeded by addition of 100 µL of DMSO to each well to solubilize the formazan crystals. It was then left in murky place for four hrs at room temprature. The absorbence was assessed at the wavelength of 595 nm using a mircotitre plate reader and results were analyzed in triplicate and the percentage was calculated.

### Gene expression analysis

Total RNA isolation, cDNA conversion and real-time PCR

The mRNA expression levels of CXCR4, HER2 were examined using real-time PCR. The sum of RNA was detached by using using a TRIR kit (Total RNA Isolation Reagent Invitrogen) and assessed spectrometrically by the method of Fourney et al. (1988) [15]. The RNA concentration was expressed in microgram (µg). Using the reverse transcriptase kit from Eurogentec (Seraing, Belgium), complementary DNA (cDNA) was synthesized from 2 µg of complete RNA as portrayed in the manufacturers' protocol. To perform real-time PCR, the reaction mixture containing 2x reaction buffer (Takara SyBr green master mix), forward and reverse primers of CXCR4 and HER2 (the primer sequences are listed in Table 1 - see PDF) in a total volume of 45 µl, the expected cDNA was made, mixed intensively, and spun down. In individual PCR vials, about 5 µl of control DNA for positive control, 5µl of water for the negative control, and 5 µl of template cDNA for samples were taken and reaction mixture (45µl) was added. 40 cycles (95°C for 5 min, 95°C for 5 sec, 60°C for 20 sec, and 72°C for 40 sec) were set up for the reaction and obtained results were plotted by the PCR machine (CFX96 Touch Real-Time PCR Detection System, USA) on a graph. Relative expression of quantity was estimated from the melt and amplification curves analysis.

### Protein expression analysis (western blotting)

The sample (50 µg) was subjected to heat denaturation at 96°C for 5 min with Laemmli buffer. Proteins were resolved by sodium dodecyl sulfate-polyacrylamide gel electrophoresis (SDS-PAGE) on 12% polyacrylamide gels and then transferred to PVDF membrane (Amersham Biosciences, UK). The membrane was blocked with 5% blocking buffer (Amersham Biosciences, UK) in TBS-T (Tris-buffered saline and Tween 20) for anhour at room temperature succeeded by incubation with primary antibody to CXCR4 and HER2 at a dilution of 1:1000. The membrane was washed thrice repeatedly with TBS- T and then incubated for an hour in horseradish peroxidase (HRP)-conjugated mouse/rabbit secondary antibody by 1:7500 dilutions in TBS-T. The membrane was again subjected to repeated wash for three times with TBS and TBS-T. Protein bands were visualized in chemidoc using enhanced chemiluminescence reagents (ECL; Amersham Biosciences, UK). The detected bands were quantified by Quantity Software (Bio-Rad). Later, the membranes were incubated in stripping buffer [50 ml, containing 62.5 mM of Tris-HCl (pH 6.7) and 1 g of SDS and 0.34 ml β-mercapto ethanol] at 55°C for 40 min. Following this, the membranes were re-probed using β-actin antibody (1:5000). In this study, a β-actin was used as loading control.

### Statistical analysis:

The obtained results from the assays were showed as mean ± SD. The Statistical evaluations were measured through a statistical package program (SPSS 10.0, IBM, Armonk, New York, United States).

## Results and Discussion:

The in vitro anticancer activity of n-hexane extract from *M. nudicaulis* in the different concentrations ranging from 3.12 to 200 µg/mL against A2780 cell lines was analyzed by cell growth inhibitory assay. Plant extract showed 86% of inhibition (Figure 1 - see PDF) at the highest 200µg/mL concentration. It possesses similar significant cell growth restriction activity in the ovarian cancer cell lines of A2780 with a low concentration (IC_50_ value) as 32.46±0.92 µg/mL, when compared with cyclophosphamide 10.17±0.53 µg/mL. The induction of cell death occurs in a shallow concentration range, like other potential anticancer drugs [16]. Thus, it may be a right candidate for an anticancer agent. The result indicates, n-hexane extract from *M. nudicaulis* holds significant anticancer activity, and it may be used to prepare good drug candidates for an anticancer agent.

**Figure 2:**
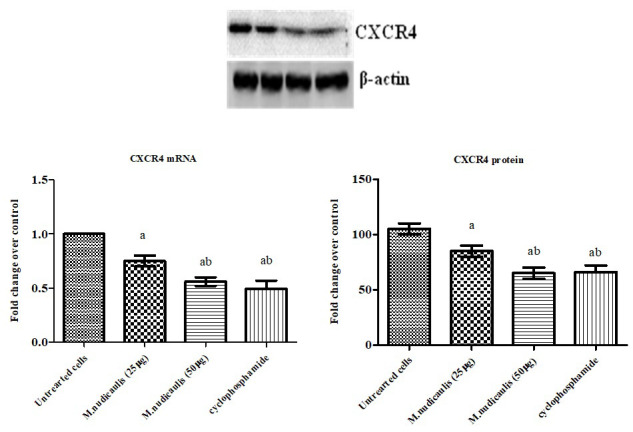
Effect of *M. nudicaulis* on CXCR4 mRNA and protein expression in A2780 human ovarian cancer cells. Cells were treated with 25 and 50µg of *M. nudicaulis* n-hexane extract for 24 h. The mRNA expression of CXCR4 mRNA gene was analyzed by real-time PCR using SYBR Green dye and protein expression by western blotting. Protein levels were quantified using densitometry analysis and are expressed in relative intensity. β-actin was used as an internal control. Target gene expression was normalized to β-actin mRNA expression and the results are expressed as fold change from control. Each bar represents mean ± SEM of 6 observations. Significance at p<0.05, a-compared with untreated control cancer cells; b-compared with 50µg *M. nudicaulis* treated A2780 cells.

**Figure 3:**
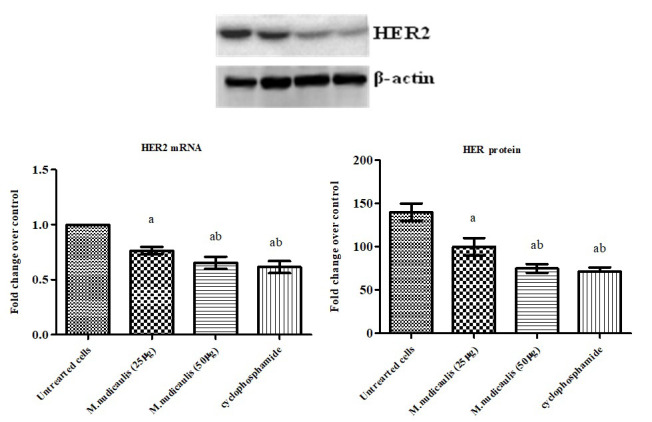
Effect of *M. nudicaulis* on HER2 mRNA and protein expression in A2780 human ovarian cancer cells. Cells were treated with 25 and 50µg of *M. nudicaulis* n-hexane extract for 24 h. The mRNA expression of HER2 mRNA gene was analyzed by real-time PCR using SYBR Green dye and protein expression by western blotting. Protein levels were quantified using densitometry analysis and are expressed in relative intensity. β-actin was used as an internal control. Target gene expression was normalized to β-actin mRNA expression and the results are expressed as fold change from control. Each bar represents mean ± SEM of 6 observations. Significance at p<0.05, a-compared with untreated control cancer cells; b-compared with 50µg *M. nudicaulis* treated A2780 cells.

Chemokine receptor 4 (CXCR4) is also recognized as a receptor for chemokine (C-X-C motif) ligand 12, a seven-transmembrane G protein-coupled receptor. A piece of accumulating evidence has shown that various types of cells, like WBCs that are produced in the lymph nodes, stem cells, the layer of cells that line heart, blood and lymphatic vessels, and membranous, flattened cells that cover the internal organs, and tumor cells, express CXCR4 [17]. In specific, advanced or metastatic ovarian cancers have been seen to control epithelial ovarian cancer (EOC) metastasis with increased rates of CXCR4 and CXCR4 / CXCL12 interactions. Nearly 59% of ovarian tumours are CXCR4-positive. Therefore, the most effective oncoprotein is the human epithelial growth factor receptor (HER, ErbB) family of receptor tyrosine kinases (RTKs). HER2 is elevated or over-expressed in multiple tumors, and is associated with unfavorable clinical results, including a strong metastasis association [18]. Furthermore, the metastatic ability of murine and human cancer cell lines is raised by HER2 [19]. Li *et al.*, (2004) found that HER2 increases the expression of CXCR4, which is also necessary for *in vitro* and *in vivo* mediated invasion by HER2 lung metastasis. HER2 also prevents ligand-induced degradation of CXCR4 [20]. Thus, we proposed that CXCR4 plays a function in metastases regulated by HER2. We analyzed the expression and protein level of CXCR4 and HER2 by RT-PCR and western blotting analysis to confirm this hypothesis. We observed that the expression of CXCR4 and HER2 in control A2780 cell lines to be intensified in Figure 2-Figure 3. In addition, the expression levels of n-hexane extracts of *M. nudicaulis* treated A2780 cells have the lowest mRNA expression and protein level provided the significant anticancer function.  The most potent antioxidant, anti-inflammatory, and anticancer activity were demonstrated by methanolic extract of *M. nudicaulis* Lam., which may cause the CXCR4 and HER2 expression controlled by *M. nudicaulis*.. It is well known that phytochemicals such as alkaloids, steroids, flavonoids, saponins, and terpenoids have the strongest anticancer effect, resulting in anticancer activity owing to *M. nudicaulis*. Also, another Mollugo sp. (pentaphylla L) showed important anti-inflammatory, antitumor and antioxidant effects in animals [11], it also indicated that n-hexane extracts of *M. nudicaulis* to produce some bioactive compounds that can regulate the expression and control inflammation of CXCR4 and HER2 expression.

## Conclusion:

This study reports that, the n-hexane extract of *M. nudicaulis* have anticancer activity with IC50 values of 32.46±0.92 µg/mL on A2780 cell lines. Moreover, CXCR4 and HER2 mRNA and protein expression were significantly reduced in *M. nudicaulis* treated A2780 cell lines. Together this study can conclude that n-hexane extract of *M. nudicaulis* holds strong antiovarian cancer activity. It might be used to develop novel drug candidates for ovarian cancer. Nevertheless, further studies are needed to authenticate the current finding.
